# Understanding the transmission dynamics of respiratory syncytial virus using multiple time series and nested models

**DOI:** 10.1016/j.mbs.2006.08.018

**Published:** 2007-09

**Authors:** L.J. White, J.N. Mandl, M.G.M. Gomes, A.T. Bodley-Tickell, P.A. Cane, P. Perez-Brena, J.C. Aguilar, M.M. Siqueira, S.A. Portes, S.M. Straliotto, M. Waris, D.J. Nokes, G.F. Medley

**Affiliations:** a Department of Biological Sciences, University of Warwick, Coventry CV4 7AL, UK; b Graduate Division of Biological and Biomedical Sciences, Emory University, Atlanta, GA 30322, USA; c Instituto Gulbenkian de Ciencia, Oeiras, Portugal; d Health Protection Agency West Midlands, Regional Surveillance Unit, 9th Floor, Ladywood House, 45 Stephenson Street, Birmingham, B2 4DY, UK; e Health Protection Agency, Porton Down, Salisbury, UK; f National Centre for Microbiology, Instituto de Salud Carlos III, Madrid, Spain; g Department of Virology, Respiratory Virus Laboratory/FIOCRUZ, Av Brasil, 4365, Rio de Janeiro, Brazil; h LACEN/FEPPS, Av Ipiranga, 5400, Porto Alegre, Brazil; i Department of Virology, University of Turku, Turku, Finland; j Centre for Geographic Medicine Research, Coast, Kenya Medical Research Institute, Kilifi, Kenya

**Keywords:** Respiratory syncytial virus, Transmission model, Immunity, Seasonality, Hospital data, Infectiousness

## Abstract

The nature and role of re-infection and partial immunity are likely to be important determinants of the transmission dynamics of human respiratory syncytial virus (hRSV). We propose a single model structure that captures four possible host responses to infection and subsequent reinfection: partial susceptibility, altered infection duration, reduced infectiousness and temporary immunity (which might be partial). The magnitude of these responses is determined by four homotopy parameters, and by setting some of these parameters to extreme values we generate a set of eight nested, deterministic transmission models. In order to investigate hRSV transmission dynamics, we applied these models to incidence data from eight international locations. Seasonality is included as cyclic variation in transmission. Parameters associated with the natural history of the infection were assumed to be independent of geographic location, while others, such as those associated with seasonality, were assumed location specific. Models incorporating either of the two extreme assumptions for immunity (none or solid and lifelong) were unable to reproduce the observed dynamics. Model fits with either waning or partial immunity to disease or both were visually comparable. The best fitting structure was a lifelong partial immunity to both disease and infection. Observed patterns were reproduced by stochastic simulations using the parameter values estimated from the deterministic models.

## 1 Introduction

Human respiratory syncytial virus (hRSV) is ubiquitous, with the great majority of people having a primary exposure before their third birthday. Infection results in lower respiratory tract disease in about 40% of primary cases and is responsible for the hospitalisation of 0.1–2% of infants each year [1]. Recurrent infections in the same individual are common, and can occur within a single epidemic as well as in subsequent epidemics [2,3]. Although re-infections are generally less severe than the primary infection [4], the relative contributions of primary infection and re-infection to hRSV transmission remains unclear.

The transmission dynamics of hRSV are strongly seasonal with a pronounced annual component in many countries. For example, in Finland, double epidemic peaks, a minor followed by a major peak, are observed every 2 years [2]. Epidemics occur each winter in many temperate climates and are often coincident with seasonal rainfall and religious festivals in tropical countries [5,6]. Although commonly ascribed to climate, the factors responsible for variation in seasonality between locations remain unproven [7–9].

Mathematical models of viral transmission are usually based on compartmentalisation of the host population based on the individual’s infection and immunity status. The two extreme models, and most studied, are the susceptible-infected-recovered (SIR) and susceptible-infected-susceptible (SIS) models. These frameworks, respectively, assume that individuals recover from infection to become either solidly immune to reinfection (e.g., as for measles, mumps and rubella), or totally susceptible to subsequent infection. An additional status is often added to the SIR framework to include those individuals who have been exposed (and are infected), but are yet to be infectious (SEIR). Intermediate models between SIR and SIS include degrees of susceptibility to reinfection, or a reinfection state that has, for example, reduced infectiousness; such a model might be susceptible-infected-(partially)-susceptible-(re)infected (SISI) [10]. Weber et al. [11] used an SEIRS model with seasonal forcing to estimate specific parameters related to seasonal transmission and acquisition of immunity. Two models of bovine RSV (bRSV) have been published: Greenhalgh et al. [12] demonstrated a subcritical endemic state (i.e., bRSV could persist in a population into which it could not invade) and de Jong et al. [13] applied a stochastic model to data. Both models were of the SISI format and therefore structurally similar to those described in other contexts [14–16]. In each of the attempts to model RSV, the model structure implicitly contains assumptions about the relationship between primary and subsequent infections. There are a number of routes by which the continuum between SIR and SIS can be bridged, which essentially depend on either considering individuals as being on a continuous scale of immunity [17] or on discretising this scale [10].

Our principal aim is to use a model in which the underlying transmission model structure can be estimated from data. We introduce a set of four ordinary differential equations that include homotopy parameters that provide paths along which different underlying transmission assumptions can be realised. In this way, we “nest” a number of biological assumptions within a single model structure. In order to gain some insight into both the underlying host–pathogen biology (which should be invariant in geographical space) and the seasonal contact pattern (which might vary between host populations), we use multiple time series from different locations to estimate parameters minimising implicit, structural assumptions. We validate the model estimates by making a quasi-independent prediction, and explore the effects of stochasticity using the best fitting model.

## 2 The data

The data sets were all records of the monthly or weekly number of diagnosed hRSV cases in a given population. In each data set these are hospitalisations, most probably predominantly of infants experiencing their first infection, representing the serious disease “tip of the iceberg” of all hRSV infections. Vaccination is not available, and so the data represent “natural” (unperturbed) infection dynamics. The eight populations from which such case notification data were acquired were:

• Porto Alegre, Brazil monthly cases from 1990 to 2003 [18]
• Rio de Janeiro, Brazil monthly cases from 1986 to 2003 [19,20]
• England and Wales monthly cases from 1991 to 1998 (Communicable Disease Surveillance Centre, UK)
• West Midlands, UK weekly cases from 1991 to 1998 (Health Protection Agency (West Midlands), Communicable Disease Surveillance Centre, UK)
• Finland monthly cases from 1981 to 2000 [2]
• Florida monthly cases from 1981 to 1997 [21]
• The Gambia monthly cases from 1990 to 1994 [11,22]
• Madrid, Spain monthly cases from 1991 to 2002 [23]
• Singapore monthly cases from 1990 to 1995 [6].

The data are shown in Figs. 3 and 4.

## 3 The model

**Fig. 1:**
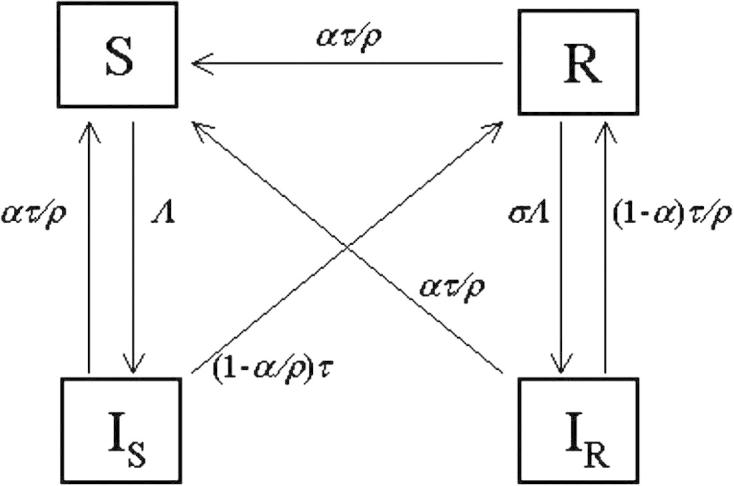
A diagram of the parent model (Model 8) of the nested set. The boxes represent the state variables of the model for each of which there is an ordinary differential equation. The arrows represent the flow between the states of the model.

The full model (within which all others are nested) is illustrated by the flow diagram in Fig. 1 with parameters as defined in Table 1. The model is developed as follows. Individuals are born at rate *μ* into the fully susceptible class (*S*) and can be infected at a rate *Λ*, wherefrom their infection (*I*_*S*_) is lost at a rate *τ*. Individuals are totally protected while they are infected, i.e., super-infection is not possible. Individuals that have been previously infected but recovered (*R*) become reinfected individuals (*I*_*R*_) at a rate *σΛ*, with potentially reduced infectiousness (by a factor *η*) and potentially reduced infectious period by a factor *ρ*, i.e., infectivity being lost at a rate *τ*/*ρ*. This structure includes two susceptible classes (*S* and *R*) and two infected classes (*I*_*S*_ and *I*_*R*_). The homotopy parameters *σ*, *η* and *ρ* determine the differences between primary and subsequent infections in terms of susceptibility, infectiousness and duration of infection, respectively.

To include temporary (waning) immunity in the same framework, we assume that all individuals with experience of infection (*I*_*S*_, *I*_*R*_, *R*) can return to the fully susceptible class (*S*). Conceptually, if the loss of immunity is a separate process to loss of infection, then individuals need not recover to a partially protected class before losing immunity. In order to preserve the previous structure, we define the rate at which immunity wanes as *ατ*/*ρ*, so that *α* = 0 defines a model in which immunity does not wane. Note that the homotopy parameters *α* and *ρ* are not strictly independent. Considering individuals with primary infection (*I*_*S*_), they recover at a total rate *τ* with a proportion *α*/*ρ* returning to the fully susceptible class and (1 − *α*/*ρ*) recovering to the partially susceptible class. Thus, to preserve the sense of the direction of flows: 0 ⩽*α* ⩽ *ρ*. The full model equations are:

$$\begin{array}{cc} \frac{dS}{dt} & =\mu P-\frac{\Lambda S}{P}+\frac{\alpha \tau }{\rho }(I_{S}+I_{R}+R)-\mu S\text{,}\\ \frac{dI_{S}}{dt} & =\frac{\Lambda S}{P}-(\tau +\mu )I_{S}\text{,}\\ \frac{dI_{R}}{dt} & =\frac{\sigma \Lambda R}{P}-\left(\frac{\tau }{\rho }+\mu \right)I_{R}\text{,}\\ \frac{dR}{dt} & =\left(1-\frac{\alpha }{\rho }\right)\tau I_{S}+(1-\alpha )\frac{\tau }{\rho }I_{R}-R\left(\frac{\sigma \Lambda }{P}+\frac{\alpha \tau }{\rho }+\mu \right)\text{.} \end{array}$$

The model variables represent the numbers of the population in each class, so that *S* + *I*_*S*_ + *I*_*R*_ + *R* = *P*, and the population size is assumed constant with birth and death rates equal. The rate of infection, *Λ*, is the product of a basic reproduction number and the total effective infectiousness: *Λ* = *Q*(*t*) × *I*(*τ* + *μ*) and *I* = *I*_*S*_ + *ηI*_*R*_. Transmission varies seasonally: $Q(t)=\frac{b\{a\cos[2\pi (t-\varphi )]+1\}}{\left(\tau +\mu \right)}$, where *b*, *a* and *φ* are treated as unknown parameters. The peak in transmission (*φ*) is given in a fraction of a year (0 is 1st January and 1 is 31st December). The relative amplitude is determined by *a*, which varies between 0 (no seasonal variation) and 1 (where transmission is 0 at its minimum and twice the mean at its maximum). The overall infectiousness is set by the transmission coefficient *b*, and defined so that the mean basic reproduction number of primary cases is given as $\overset{¯}{Q}=\frac{b}{\left(\tau +\mu \right)}$.

The four homotopy parameters (*σ*, *ρ*, *η* and *α*) take values between 0 and 1, and by setting some of them to the extremes and estimating others, we obtain different sub-models. The full model without seasonality has a backwards bifurcation for certain conditions on the parameters, however, this condition does not apply for realistic parameter value ranges for hRSV.

### 3.1 Model parameterisation

Eight combinations of global and estimated parameters were the foundation of the analysis (Table 2).

#### 3.1.1 Global parameters

Parameters not permitted to vary between locations are described as global (Table 2). The average duration of primary infection is fixed (i.e., not estimated) at 9 days for all models and locations [4]. The homotopy parameters are treated as global, in that they define the biological interaction between the host immune response and the virus. In Model 1, the parameters *σ*, *ρ*, *η* and *α* are fixed at values that make the model a standard SIS model. In this case, immunity does not wane, and there are no transmission differences between the susceptibility classes and infection classes. In Model 2, the parameters *σ*, *ρ*, *η* and *α* are fixed at values that make the model a standard SIR model. In Model 3, the parameters *σ*, *ρ* and *η* are fixed at values that make the model a standard SIRS model. The parameter *α* is estimated to calculate a global value of the average duration of immunity. In Model 4, the parameters *ρ*, *η* and *α* are fixed at values that make the model equivalent to the partial immunity model developed by Gomes et al. [10]. The parameter *σ* is estimated to obtain a global value of the altered susceptibility factor. In Model 5, the parameters *ρ* and *η* remain fixed while the partial immunity is allowed to wane. The parameters *σ* and *α* are estimated to obtain global values of the altered susceptibility factor and average duration of immunity respectively. In Model 6, the parameter *α* is fixed at a value that makes the model equivalent to that proposed by Greenhalgh et al. [12]. The parameters *σ*, *ρ* and *η* are estimated to obtain global values of the altered susceptibility, altered duration of infection and altered infectiousness factors, respectively. Model 7 assumes temporary resistance to disease rather than infection. The parameter *σ* is fixed at unity while the parameters *ρ*, *η* and *α* are allowed to assume some value between zero and unity. This model therefore defines partial temporary immunity as an altered response to subsequent infection given that challenge occurs within a given time since previous infection. Model 8 is an extension to that proposed by Greenhalgh et al. [12] that allows the partial immunity to wane. The parameters *σ*, *η*, *ρ* and *α* are estimated to obtain global values of the altered susceptibility factor, altered infectiousness factor, altered duration of infection factor and average duration of immunity, respectively.

A further global parameter is the percentage of primary cases that are hospitalised, *h*. The models predict the numbers of the population in each state at a given time (in years), and the data give the new hospitalised cases per month, therefore we must use a scaling factor in order to compare the model output with the data. Weber et al. [11] assumed that the incidence rate (number of new cases per unit time) is proportional to the number in the infected class at that time. In fitting the models, we assume that the data represent a proportion of the incidence of infections of completely susceptible individuals, i.e., a proportion of *ΛS*. The observed data are the cumulative numbers of new cases over an interval (Δ*t*), and if only a percentage *h* of primary cases are hospitalised, then the observed number of cases, *D*, will be

$$D=\frac{h}{100}\int_{\Delta t}\Lambda S\text{.}$$

If we assume that the incidence of hospitalised infections is constant over the interval, then the model output must be multiplied by the following scaling factor to relate to data

$$\frac{D}{\Lambda S}=\frac{h}{100}\Delta t\text{.}$$

The value of *h* was estimated for Finland as 2.1 [24]. In the Gambia, the RSV hospitalisation rate per child year was estimated at 0.0087 [25], which if it is assumed that all children will have experienced an infection by the age of 2 years would give a value of *h* of 1.74. This publication also cites a range of 0.018–0.198 per child year for RSV hospitalisation rates in developing countries. A recent community study in Kenya [1] stated that of 338 children, 133 tested positive for RSV and 4 were hospitalised with severe RSV. However the test used for RSV infection is known not to be 100% sensitive, so a minimum estimate of *h* would be 4/338= 0.012 and a maximum estimate of *h* would be 4/133 = 0.03. Since the range of estimates for *h* for developing countries encompasses the range of estimates for developed countries, we will assume that it is a global parameter in the estimation process.

### 3.2 Location specific parameters

For each of the eight locations, estimates were obtained for: (i) mean basic reproduction numbers (*R*_0_); (ii) relative amplitudes of seasonal variation of the transmission rate (*a*); (iii) times in the year of the peak of the transmission rate (*φ*).

### 3.3 Modelling fitting

The negative log-likelihood, LL_−_, was calculated under the assumption that the observations follow a Poisson distribution as follows:

$$\text{for}\;n_{i}\in \{1\text{,}..\text{,}N_{i}\}\text{,}{\text{LL}}_{n_{i}}=d_{n_{i}}\ln(D_{n_{i}})-D_{n_{i}}-\overset{d_{n_{i}}}{\underset{j=1}{\sum }}\ln(j)\text{,}$$

$${\text{LL}}_{-}=-\overset{8}{\underset{i=1}{\sum }}\overset{N_{i}}{\underset{n_{i}=1}{\sum }}{\text{LL}}_{n_{i}}\text{,}$$

where *N*_*i*_ is the number of observations in location *i*, $d_{n_{i}}$ are the individual observations and $D_{n_{i}}$ are the corresponding expected incidences.

The Poisson distribution was used because it is most suitable for considering count data [26]. This definition of the likelihood is also intrinsically weighted for larger population sizes and higher numbers of samples.

The negative log-likelihood, LL_−_, was used in the optimise feature of the computer package Berkeley Madonna [27] to obtain the best fit of each model to the eight data sets simultaneously. The fits of nested models were compared using the likelihood ratio. In each case the stable limit cycle of the model was fitted to the data, we assume a stable limit cycle. There is no reason to assume that the systems were perturbed at the time of the beginning of observation in any location and therefore be in a transient phase. What is more, the assumption of transient dynamics would require the estimation of initial conditions for the variables of each model therefore increasing the number of unknown parameters unnecessarily. The best fitting initial conditions were for a condition very close to stable limit cycles, therefore justifying their use. Thus an assumption of transient dynamics would allow a slightly higher and unjustified contribution of the earlier parts of the data sets to the likelihood.

In order to increase the accuracy of the model fit, multiple optimisations were carried out. First we obtained a visual fit using multiple runs and fits of nested models if available. We then performed minimisation of the negative log-likelihood using initial guesses that encompassed the visually fitted values. We repeated the minimisation using initial guesses that encompassed the previous fitted values. We repeated the previous step until each resulting estimate was within two significant figures of its initial guesses. This process was completed with between 12 and 21 minimisations.

**Fig. 2:**
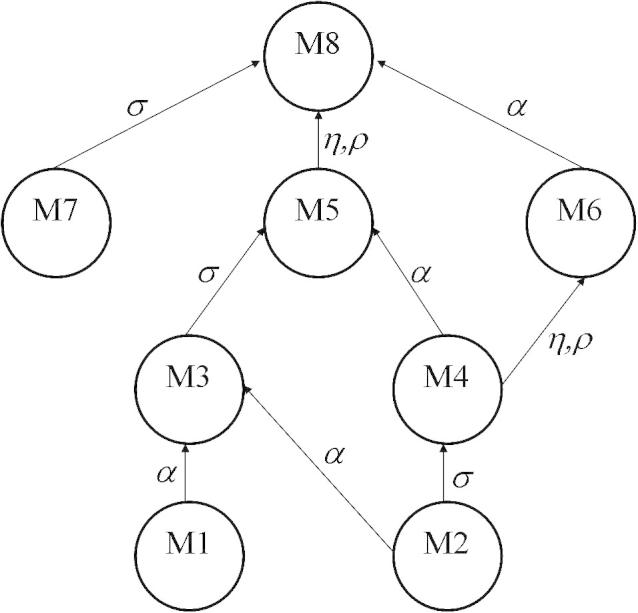
A diagram showing the relationships of the nested models. An arrow from Model A to Model B indicates that A can be transformed to B by explicitly including the labelled parameters.

The diagram in Fig. 2 shows the nested structure of the set of 8 models. The diagram demonstrates the fact that although all the models are nested within Model 8, they cannot be organised as a set of nested models with each model being nested within the next up to Model 8. Therefore some pairs of models cannot be compared directly using their likelihood ratio (e.g., Models 6 and 7), but all models can be compared with Model 8.

### 3.4 Stochastic model

The model equations were discretised using the Euler approximation for a differential and an interval of 0.1 month. The difference equations are as follows.

$$\begin{array}{cc} S_{n_{i}}^{\prime }= & \mu_{i}P_{i}-C_{n_{i}}S_{n_{i}}(I_{S\text{,}n_{i}}+\eta I_{R\text{,}n_{i}})+\frac{\alpha \tau }{\rho }\max[(P_{i}-S_{n_{i}})\text{,}0]-\mu_{i}S_{n_{i}}\text{,}\\ I_{S\text{,}n_{i}}^{\prime }= & C_{n_{i}}S_{n_{i}}(I_{S\text{,}n_{i}}+\eta I_{R\text{,}n_{i}})-(\tau +\mu_{i})I_{S\text{,}n_{i}}\text{,}\\ I_{R\text{,}n_{i}}^{\prime }= & \sigma C_{n_{i}}(1-S_{n_{i}}-I_{S\text{,}n_{i}}-I_{R\text{,}n_{i}})(I_{S\text{,}n_{i}}+\eta I_{R\text{,}n_{i}})-\left(\frac{\tau }{\rho }+\mu_{i}\right)I_{R\text{,}n_{i}}\text{,}\\ C_{n_{i}}= & B_{i}\{a_{i}\cos[2\pi (t_{n_{i}}-\varphi_{i})]+1\}\text{,}\\ S_{1_{i}}= & S_{0i}\text{,}\\ S_{n_{i}+1}= & \max\{\Theta [S_{n_{i}}+S_{n_{i}}^{\prime }(t_{n_{i}}-t_{n_{i}-1})]\text{,}0\}\text{,}\\ I_{S\text{,}1_{i}}= & I_{S_{0i}}\text{,}\\ I_{S\text{,}n_{i}+1}= & \max\{\Theta [I_{S\text{,}n_{i}}+I_{S\text{,}n_{i}}^{\prime }(t_{n_{i}}-t_{n_{i}-1})]\text{,}0\}+\Theta [\xi P_{i}]\text{,}\\ I_{R\text{,}1_{i}}= & I_{R_{0i}}\text{,}\\ I_{R\text{,}n_{i}+1}= & \max\{\Theta [I_{R\text{,}n_{i}}+I_{R\text{,}n_{i}}^{\prime }(t_{n_{i}}-t_{n_{i}-1})]\text{,}0\}+\Theta [\xi P_{i}]\text{,}\\ D_{m}= & C_{n_{i}}S_{n_{i}}(I_{S\text{,}n_{i}}+\eta I_{R\text{,}n_{i}})\frac{h}{100}\frac{1}{12}\text{,}\;m=n\;\text{if}\;n\in Z \end{array}$$

where *Θ*[*x*] is sampled from a Poisson distribution of mean *x*. The mean number of individuals in a given state (e.g., *I*_*S*_) at a given time is determined from the previous numbers of individuals in each state and the parameters of the model. Then a value is sampled from a Poisson distribution with that mean. In order to avoid fade-out of infection during epidemic troughs, a random number of infections is introduced (sampled from a Poisson distribution of mean *ξ*P_*i*_) representing a number of imported infections from outside the population of location *i*.

## 4 Results

### 4.1 Parameter estimation and model comparison

**Fig. 3:**
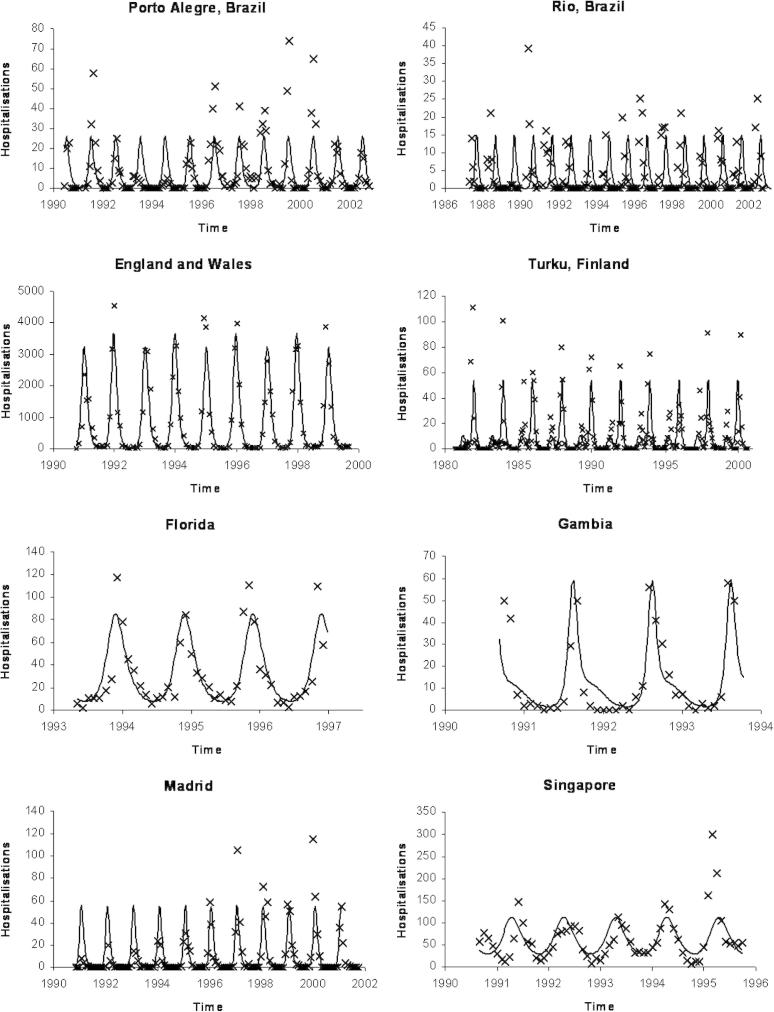
The fit of Model 6 to the eight data sets. For each location, the model prediction of the incidence (line) is plotted with the monthly cases for that location (crosses).

The parameter estimates are given in Tables 2 and 3 and summaries of fits and derived parameters are given in Table 4. Those models that were nested were compared using the *χ*^2^ distribution and the results are shown in Table 5. It is clear that the simplest, best fitting model is Model 6. The fit of Model 6 to the data sets is shown in Fig. 3.

The estimates for Model 6 would indicate that once infected with RSV, a person will remain partially immune for their lifetime. This partial immunity would take the form of a reduced risk of infection (68%) when re-challenged with reinfections being less infectious (25%) and lasting for about half the time (60%) compared with a primary infection.

From Table 4, it can be seen that Models 1 and 2 fit poorly compared to the other models. Of the remaining models, Models 3, 5 and 7 require the percentage of reported cases to be much lower than for Models 6 and 8. This is because these models include a loss of immunity and therefore a much larger proportion of the population would inhabit the primary infected class at any time and would include all ages rather than just infants undergoing their first infection. This however is not the case for Models 4, 6 and 8, where there is no return to the totally susceptible state after recovery from infection. Thus for these models, the predicted percentage of reported primary cases is consistently estimated at 1.3%.

#### 4.1.1 Quasi-independent validation

**Fig. 4:**
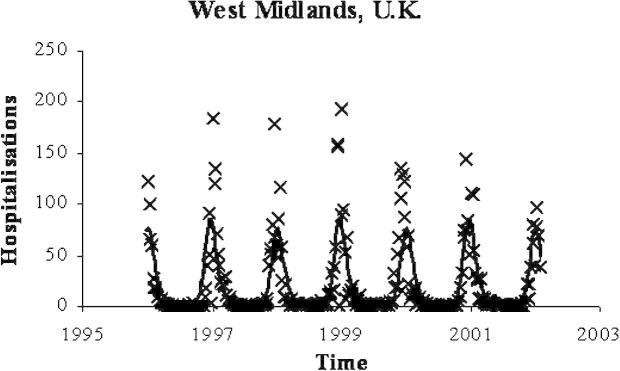
West Midlands RSV data with Model 6 prediction. The model prediction of the incidence (line) plotted with the weekly cases from the West Midlands (crosses).

The fitted global and local parameters for England and Wales from Model 6 were then used to reproduce the weekly incidence of hRSV in the West Midlands region of England. Since the population of the West Midlands is roughly a tenth (0.100775) of that in England and Wales (ONS, Population Estimates Unit), the population size and inter-sampling interval for England and Wales was altered accordingly and the incidence from Model 6 was plotted against the hRSV case data for the West Midlands in Fig. 4. The model prediction underestimates the height of the peaks, but replicates the timing and shape of the annual epidemics.

#### 4.1.2 Stochastic simulation

**Fig. 5:**
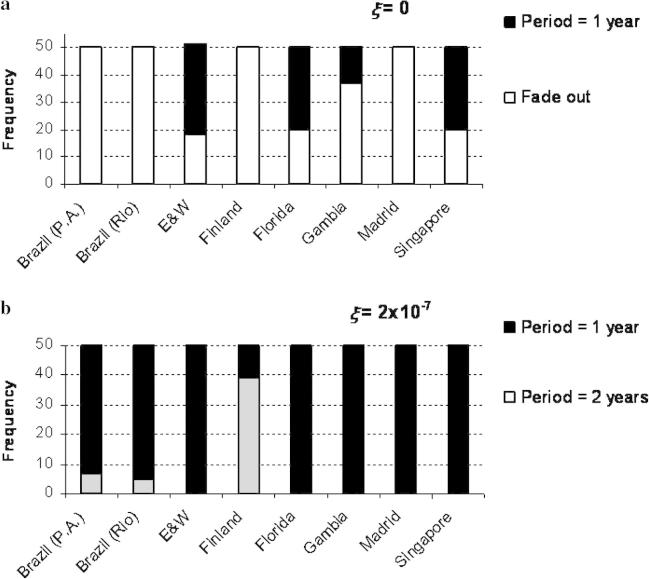
Bar charts showing the results of 50 simulations of the stochastic version of Model 6 for (a) closed populations (*ξ* = 0) and (b) populations where there is random influx of infections (*ξ* = 2 × 10^−7^).

The stochastic model was run for each population for a period of 20 years. Fifty runs were performed for two values of *ξ* (0 and 2 × 10^−7^). If it is assumed that each population is closed and that infections can only arise from transmission from other members of that population, i.e., that *ξ* = 0, then fade out can occur very easily during simulation (Fig. 5(a)).

When some infection events were permitted to occur randomly and independently of the infection levels in the population (i.e., *ξ* > 0), simulations then showed no fade out and rarely cycled at a different periodicity from the observed data for *ξ* = 2 × 10^−7^ (Fig. 5(b)), which indicates a random influx of RSV cases at the level about one millionth of the population each week into each infectious class. Increasing this value further forced oscillations into a period of one year in all regions and increased the value of the inter-epidemic troughs.

Stochastic versions of Models 3, 5 and 7 were also run and had similar characteristics to Model 6, although fade out was more common.

## 5 Discussion

We have used a dynamic model with four homotopy parameters to gain insight into the natural history of hRSV infection and reinfection. By applying a family of nested models to multiple data sets we are able to discount the SIR model (Model 2), which assumes that immunity is solid and lifelong. This is an expected result since reinfection is known to occur [28] and some individuals regaining susceptibility is not unexpected given that RSV has a high level of antigenic diversity, and this diversity is known to change with time [5,29,30]. However, we can also discount the simplest structures for reinfection, i.e., where reinfection is identical to primary infection (Models 1 and 4) although the risk of reinfection may be comparatively lower (Model 4). The remaining models provided comparable visual fits, but Model 6 was found to provide the best and most parsimonious fit based on statistical inference of the calculated log-likelihoods.

A breakdown of the negative log-likelihood by data set (see Table 4) for each model indicates that most between-model variation occurs for the Finland data set although there is noticeable variation in the Gambian values as well. There was a higher negative log-likelihood than for Models 6 and 8 for all other models for Finland. Since the dynamics for Finland are more complex with a biennial signal rather than an annual signal, it is reasonable to expect more variation in these results. However, Model 6 was one of the two best fitting models for every data set, indicating that the fit was not completely dominated by the Finland data.

The fitting procedure for the parent model (Model 8) involves maximising the likelihood in 35-dimensional parameter space. This may seem a very high number of parameters, but it is important to note that many of these parameters are local and are associated with specific independent data sets. Therefore the parameter space is structured and is more like six 4-dimensional spaces and two 3-dimensional spaces all linked by a further 5 dimensions. However, an improved fitting method would be to use a multi-dimensional algorithm for each set of local parameters coupled with an update of the global parameters for each iteration, until convergence is achieved.

**Fig. 6:**
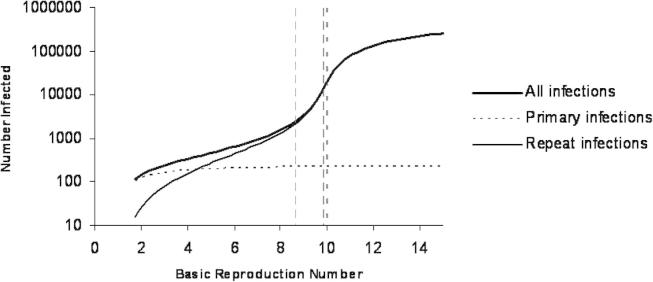
Graph showing the predicted number of (primary and repeat) infections by Model 6 without seasonal forcing using parameter values estimated from the Finland data set for different values of basic reproduction number including the reinfection threshold (bold dotted) and the estimated lower and upper seasonal limits (dashed).

Model 6 describes a structure where immunity is partial but lifelong. Partial immunity manifests in two ways: first, a reduced risk of infection (partial immunity to infection) and second, an altered infectiousness and duration of repeat infections (partial immunity to disease). Interestingly, of the visually well fitting models, this is the only model that does not include any return of hosts to the completely naïve, fully susceptible state. This permits a decomposition into two transmission submodels: fully susceptible hosts (*S*) become infected (*I*_*S*_) at a rate *Λ* to recover with partial immunity; partially immune hosts (*R*) become infected (*I*_*R*_) at a reduced rate *σΛ* to recover back into *R* generating a recurrent process. The first submodel induces the epidemic threshold (*R*_0_ = 1) above which infection invades a host population, and the second determines the reinfection threshold (*R*_0_ ≅ 1/*σηρ* ≅ 10, for model 6 with parameters as in Table 2). The estimated mean basic reproduction number for this model is consistently estimated at about 9.3, so that the seasonal changes in transmission oscillate near the threshold. For example, the basic reproduction number for Finland varies between 8.6 and 9.8 and results in a dramatic change in infection prevalence (Fig. 6).

Although Models 6 and 8 were found to be the best fitting models in the deterministic context, it is important to note that Models 3, 5 and 7 also provided good visual fits and their stochastic versions reproduced the observed dynamics. The fitted homotopy parameter values in Table 2 suggest that these three models are very similar. Hence, there appear only two model forms supported by the data: (i) lifelong partial immunity, and (ii) waning immunity. It should be possible to investigate which of the model types is most plausible through epidemiological investigations of individuals with known history of infection, and such studies are currently on going in Kenya [31] and India [32]. Evidence exists for reduced levels [33] and durations [33–35] of viral shedding during infection of adults and older children compared with young children [34,36]. This appears to support a lifelong alteration of the response to reinfection as indicated by Model 6. The estimated amplitudes for the oscillations in the transmission coefficient reveal different patterns under different model types (Table 4). Therefore, understanding what underlies seasonality can provide another useful indicator for model plausibility.

The predicted percentage of primary infections that are hospitalised is estimated at 1.3% for the partial immunity model (6) which is not dissimilar to a previous estimate of this value of 2.45% [11]. It is estimated at 0.038% for the waning immunity models (3, 5 and 7). This is because individuals in these models are able to completely lose their immunity and thus experience a primary infection more than once and at any age. However, hRSV infections of individuals older than 2 or 3 years that result in hospitalisation are negligible in number [37]. Therefore, given the assumption that the populations are uniformly distributed over age, the estimated percentage of primary cases (as defined by the waning immunity models) should be roughly the estimate for the partial immunity model divided by the average life expectancy (about 70 years) and multiplied by the average age of hospitalisation (about 2 years). This is in fact the case here since $\frac{1.3\times 2}{0.038}≅70$. It could also be the case that the proportion hospitalised is dependent on the state of the epidemic, acting through, for example maternal antibody or dose effects.

De Jong et al. [13] showed that infections in previously infected (seropositive) animals are not likely to be the cause of persistence of bRSV in dairy farms. We found that persistence was common in three of the eight regions from stochastic simulation, and that persistence was only achieved in the remaining five locations if some amount of infections could be introduced. Some influx of infection from outside each location would seem a reasonable assumption, since none of the locations are completely isolated from other areas where hRSV is present.

We must be somewhat distrustful of the estimated parameter values given here due to the lack of independent estimates for the population sizes for all but the England and Wales and Turku (Finland) data sets. A recent identifiability analysis, performed on an SIR model with seasonal forcing (similar to Model 2) proved that the model was unidentifiable if either the population size or percentage hospitalised was unknown [38], so that even with perfect, continuous, noise-free data for the transient dynamical phase, unique estimates for the parameter values cannot be obtained. However, we believe that our results pertaining to model structure are robust since the two known population sizes will provide constraints on the global parameters (including the percentage of primary cases hospitalised) and these will in turn constrain the estimates for the remaining population sizes.

The advantage of a comparative analysis of global data is the investigation of common determinants of transmission. If temperature variation were the explanation for the seasonality of hRSV globally, we might expect the ordering of the locations by amplitude to correlate with the ordering according to latitude, which is not the case. Likewise, there is no consistent pattern relating the timing of peak transmission with temperature change. There are many apparent patterns in results in Table 4, however the number of global locations is too small to be confident of proposing robust hypotheses. We suggest that more likely explanations of epidemic patterns in hRSV disease will involve aspects of spatial dynamics, genetic diversity (acting through susceptibility to reinfection) as well as currently undefined seasonal signals.

hRSV exhibits substantial genetic variation, that has been linked to the risk of reinfection, i.e., the possibility of is genotype specific immunity. The simple model structures used here include genetic variation implicitly in the process of acquisition and loss of immunity, for example partial immunity to reinfection could be due to challenge with a different (heterologous) strain. The interactions between genetics and dynamics have been explored for a subset of the data presented here coupled with typing data [39], and demonstrate that these interactions are able to produce complex dynamics (e.g., 6 point cycles). However, the underlying seasonality remains largely unexplained.

The general condition for an epidemic is that the product of the density of susceptibles and basic reproduction number (the effective reproduction number) be greater than unity. Seasonality in SIR infections (e.g., measles) has been explained by the timing of the school year (i.e., when contact rates between children substantially increase). Although measles and hRSV generally have different seasonal patterns, we suggest that both patterns could be due to the same seasonal forcing, given that the duration of immunity against measles is different from that against hRSV. Thus, it could be that the same seasonal factors drive transmission patterns of hRSV, measles and other viruses, but the variation in timing of observed incidence is due to differences in immunity. We plan to explore this further with a number of viral infections.

## Figures and Tables

**Table 1:** Parameter definitions

	Parameter	Description
Homotopy parameters	*σ*	Partial susceptibility factor
	*ρ*	Factor reducing the duration of infection
	*η*	Factor reducing infectiousness
	*α*	Waning immunity coefficient
		
Seasonal transmission parameters	*a*	Relative amplitude of seasonal variation of transmission
	*b*	Average transmission coefficient
	*φ*	Time in the year of the peak of the transmission
		
Rate parameters	*μ*	Birth and death rate
	*τ*	Rate of loss of primary infection
		
Scaling parameters	*h*	Percentage of primary infections that are hospitalised

**Table 2:** Model definitions and global parameter estimates

Model	*s*	*r*	*h*	*a*	% of reported primary cases	Duration of immunity (years)	*t* (year^−1^)	Comments on model
1	**0**	**1**	**1**	**1**	0.0084	**0**	**40.56**	Susceptible-infected-susceptible (SIS)
2	**0**	**1**	**1**	**0**	1.2	**Lifelong**		Susceptible-infected-resistant (SIR)
3	**0**	**1**	**1**	0.046	0.041	0.54		Susceptible-infected-resistant-susceptible (SIRS)
4	0.01	**1**	**1**	**0**	1.3	**Lifelong**		Partial immunity
5	0.00015	**1**	**1**	0.043	0.042	0.57		Waning partial immunity
6	0.68	0.25	0.60	**0**	1.3	**Lifelong**		Lifelong partial immunity with altered secondary infections
7	**1**	1.0	0.043	0.046	0.041	0.54		Immunity (that wanes) in the form of altered secondary infections
8	0.68	0.25	0.59	1.1 × 10^−6^	1.3	5400 (i.e., lifelong)		Waning partial immunity with altered secondary infections

Parameter values in bold are fixed (not fitted).

**Table 3:** Location specific parameter estimates

Region	*μ*	Population size							
		Model							
		1	2	3	4	5	6	7	8
Bra.(PA)	**0.0145**	3.7 × 10^5^	8.7 × 10^5^	3.5 × 10^5^	5.2 × 10^5^	4.5 × 10^5^	4.4 × 10^5^	3.5 × 10^5^	4.6 × 10^5^
Bra.(Rio)	**0.0145**	2.6 × 10^5^	3.2 × 10^5^	1.2 × 10^5^	1.8 × 10^5^	2.0 × 10^5^	2.1 × 10^5^	1.2 × 10^5^	2.3 × 10^5^
E. and W.	**0.014**	**5.7** × **10**^**7**^	**5.7** × **10**^**7**^	**5.7** × **10**^**7**^	**5.7** × **10**^**7**^	**5.7** × **10**^**7**^	**5.7** × **10**^**7**^	**5.7** × **10**^**7**^	**5.7** × **10**^**7**^
Finland	**0.012**	**7.5** × **10**^**5**^	**7.5** × **10**^**5**^	**7.5** × **10**^**5**^	**7.5** × **10**^**5**^	**7.5** × **10**^**5**^	**7.5** × **10**^**5**^	**7.5** × **10**^**5**^	**7.5** × **10**^**5**^
Florida	**0.016**	4.5 × 10^6^	1.9 × 10^6^	1.2 × 10^6^	1.9 × 10^6^	2.1 × 10^6^	2.0 × 10^6^	1.2 × 10^6^	2.0 × 10^6^
Gambia	**0.045**	5.4 × 10^5^	2.9 × 10^5^	8.0 × 10^5^	3.2 × 10^5^	1.3 × 10^6^	2.8 × 10^5^	8.0 × 10^5^	2.9 × 10^5^
Madrid	**0.012**	6.7 × 10^5^	8.8 × 10^5^	6.4 × 10^5^	7.2 × 10^5^	5.8 × 10^5^	8.7 × 10^5^	6.4 × 10^5^	8.5 × 10^5^
Singapore	**0.016**	1.9 × 10^6^	4.0 × 10^6^	2.2 × 10^6^	3.7 × 10^6^	2.0 × 10^6^	3.7 × 10^6^	2.2 × 10^6^	3.5 × 10^6^

Parameter values in bold are fixed.

**Table 4:** Estimated transmission parameters and model fits

Regions	Models							
	1	2	3	4	5	6	7	8
*Average basic reproduction number of primary cases (Q)*								
Brazil (P.A.)	1.09	67	1.5	58	1.3	9.4	1.5	9.4
Brazil (Rio)	1.05	79	1.7	67	1.3	9.3	1.7	9.4
E&W	1.06	64	1.3	53	1.3	9.3	1.3	9.4
Finland	1.06	69	1.2	52	1.3	9.2	1.2	9.3
Florida	1.03	52	1.7	30	1.3	9.4	1.7	9.3
Gambia	1.11	42	1.4	30	1.2	9.4	1.4	9.4
Madrid	1.06	67	1.3	61	1.4	9.4	1.3	9.5
Singapore	1.15	49	1.7	48	1.9	9.4	1.8	9.4
								
*Amplitude (a)*								
Brazil (P.A.)	0.35	0.05	0.13	0.13	0.19	0.07	0.13	0.06
Brazil (Rio)	0.50	0.07	0.22	0.19	0.23	0.09	0.22	0.11
E&W	0.39	0.12	0.26	0.18	0.24	0.09	0.26	0.09
Finland	0.15	0.32	0.27	0.26	0.27	0.07	0.27	0.07
Florida	0.18	0.05	0.10	0.12	0.11	0.06	0.10	0.04
Gambia	0.38	0.21	0.12	0.09	0.16	0.10	0.12	0.10
Madrid	0.57	0.15	0.30	0.24	0.29	0.12	0.29	0.12
Singapore	0.13	0.03	0.06	0.04	0.07	0.02	0.07	0.02
								
*Timing of peak transmission* (*φ*)								
Brazil (P.A.)	0.39	0.44	0.43	0.44	0.35	0.40	0.43	0.39
Brazil (Rio)	0.16	0.30	0.35	0.36	0.18	0.22	0.36	0.22
E&W	0.83	0.84	0.26	0.86	0.83	0.84	0.83	0.84
Finland	0.59	0.79	0.66	0.78	0.67	0.70	0.66	0.71
Florida	0.72	0.73	0.96	0.73	0.71	0.80	0.96	0.78
Gambia	0.55	0.83	0.56	0.77	0.48	0.71	0.56	0.71
Madrid	0.89	0.92	0.92	0.95	0.91	0.92	0.92	0.92
Singapore	0.19	0.19	0.40	0.22	0.46	0.18	0.40	0.20
								
*Negative log-likelihood* (LL_−_)								
Brazil (P.A.)	699	878	700	690	703	690	710	698
Brazil (Rio)	420	518	440	449	429	414	433	417
E&W	9766	9846	8640	9226	8755	8348	8633	8357
Finland	2659	1664	1784	1214	1633	1361	1827	1344
Florida	245	270	236	243	293	236	240	239
Gambia	202	239	206	196	213	190	208	182
Madrid	637	698	645	643	633	643	656	640
Singapore	798	814	814	806	822	807	811	810
**Total**	**15425**	**14927**	**13465**	**13466**	**13342**	**12689**	**13517**	**12686**

**Table 5:** Model comparisons

	7	6	5	4	3	2	1
8	0	0.0143059	0	0	0	0	0
7		NN	NN	NN	NN	NN	NN
6			NN	0	NN	0	NN
5				0	0	0	0
4					NN	0	NN
3						NN	0

NN indicates that the models are not nested and therefore cannot be compared.
